# High-Temperature Synthesis of Ordered Mesoporous Aluminosilicates from ZSM-5 Nanoseeds with Improved Acidic Properties

**DOI:** 10.3390/nano4030712

**Published:** 2014-08-18

**Authors:** Xuan Hoan Vu, Reinhard Eckelt, Udo Armbruster, Andreas Martin

**Affiliations:** Leibniz-Institut für Katalyse e.V. an der Universität Rostock, Albert-Einstein-Straße 29a, 18059 Rostock, Germany; E-Mails: hoan.vu@catalysis.de (X.H.V.); reinhard.eckelt@catalysis.de (R.E.); udo.ambruster@catalysis.de (U.A.)

**Keywords:** zeolite nanoseeds, SBA-15 analogs, cumene cracking, acidity, hydrothermal stability

## Abstract

Ordered mesoporous SBA-15 analogs with different Si/Al ratios were successfully prepared in a two-step process from self-assembly of ZSM-5 nanoseeds at high temperature in mildly acidic media (473 K, pH 3.5). The obtained products were characterized as SAXS, XRD, N_2_ sorption, FTIR, TEM, NH_3_-TPD, AAS and ICP. The results show that the initial Si/Al molar ratio of ZSM-5 precursors strongly affects the final materials’ properties. A highly condensed, well-ordered mesoporous SBA-15 analog with improved hydrothermal stability and acidic properties can be prepared from low aluminum containing ZSM-5 precursors (Si/Al ≥ 20). Reducing the initial Si/Al molar ratio to 10, however, leads to the formation of a disordered mesoporous SBA-15 type material accompanied by degraded textural and acidic properties. The gas phase cracking of cumene, carried out as probe reaction to evaluate Brønsted acidity, reveals that an increased density of Brønsted acid sites has been achieved over the SBA-15 analogs compared to conventional Al-SBA-15 due to the preservation of zeolite building units in the mesopore walls of the SBA-15 analogs.

## 1. Introduction

Zeolites are a unique class of crystalline aluminosilicates that are widely used as catalysts in oil refining and petrochemical industry. The exceptional performance of zeolites primarily stems from their strong Brønsted acidity, hydrothermal stability and uniform micropores of molecular dimensions (typically 0.25–1 nm) [1,2]. Despite their great success, zeolite based catalysts have one main drawback. As one side effect of micropores, zeolites often suffer from internal diffusion limitations particularly when large molecules are involved. Thus, the efficiency in conversion of bulky molecules, e.g., heavy oils might be low because of the restricted access to pores and the slow molecular transport to and from active sites of zeolites. In order to overcome these mass transfer limitations, ordered mesoporous materials (OMMs) with regular mesopores in the range of 2–30 nm have been developed. The diffusion regime in OMMs is typically Knudsen diffusion, whose diffusivity is several orders of magnitude higher than that of “configurational” diffusion inside micropores of zeolites [3]. Unfortunately, OMMs fail to mimic the key characteristics of zeolites, *i.e.*, strong intrinsic acidity and high hydrothermal stability, owing to the non-crystalline nature of mesopore walls, which severely hinders their practical applications. Accordingly, the introduction of zeolite building units into mesopore walls by using zeolite seeds as building block to construct a mesostructure in a two-step process has been extensively studied [4,5]. 

Zeolite nanoseeds, also known as precursors or protozeolitic nanoparticles which are presumed to consist of zeolite building units, can be prepared by shortening the hydrothermal treatment time required for evolution of classical zeolite crystals. The use of zeolite precursors for the assembly with triblock copolymer Pluronic P123 as a structure directing agent in strongly acidic media has led to the formation of ordered mesoporous SBA-15 analogs with improved hydrothermal stability and acidic properties [4,5,6,7,8]. The reason for such improvements is not clear, but the retention of zeolite building units in the mesopore walls is thought to play an important role. However, most of these studies were carried out in strongly acidic media at mild temperatures (typically 373–423 K), which resulted in low incorporated aluminum and imperfectly condensed mesopore walls. Consequently, the hydrothermal stability and acidic properties of the known SBA-15 analogs, e.g., MAS-9 [6] or MSU-S/H [8] are still inferior to those of zeolites, being unsatisfactory for industrial applications. Recently, we developed an effective method for further improving the hydrothermal stability and acidic properties of ordered mesoporous SBA-15 analogs assembled from ZSM-5 precursors (denoted as SAZ) by high-temperature synthesis and pH adjustment [9]. The ordered mesostructure of SAZ materials was well preserved even upon steaming at 1073 K for 4 h with little loss in the specific BET surface area by 11% only, while their acidic properties were substantially enhanced in terms of both number and strength of acid sites.

In this study, the effect of the initial Si/Al molar ratio of ZSM-5 nanoseeds on the final materials properties is investigated in an attempt to increase incorporated aluminum, and thereby the acid site density of SAZ materials while retaining their mesostructural ordering. To achieve this goal, the ZSM-5 nanoseeds with different Si/Al molar ratios ranging from 10 to 50 are prepared and added to the surfactant P123 solution for the self-assembly of a mesostructure under the optimized conditions [9]. After thorough characterizations by various techniques, the gas phase cracking of cumene was performed as probe reaction to evaluate the Brønsted acidity of SAZ materials compared to that of Al-SBA-15 and H-ZSM-5.

## 2. Results and Discussion

### 2.1. Physicochemical Characterization

Table 1 gives the elemental composition of SAZ materials, Al-SBA-15 and H-ZSM-5 analyzed by inductively coupled plasma atomic emission spectroscopy (ICP-AES) and atomic absorption spectroscopy (AAS). Most of aluminum in the initial gel of ZSM-5 nanoseeds has been successfully incorporated in the final SAZ solids. For example, the final Si/Al ratio of SAZ-20 is 23, which is close to 20 of the initial Si/Al ratio in the gel. This further confirms the beneficial role of pH adjusting method in introducing aluminum in SBA-15 type materials [9,10,11]. Compared to SAZ samples, Al-SBA-15 prepared from conventional silica based sources (TEOS) has a relatively higher Si/Al ratio than that of the initial gel mixture, showing the advantages of zeolite nanoseed precursors over conventional ones, being consistent with the earlier reports [6,7,8].

**Table 1:** Physicochemical properties of SAZ materials, Al-SBA-15 and H-ZSM-5.

Sample ^a^	Initial Si/Al ^d^	Final Si/Al ^e^	d_100_ (nm)	a_0_ (nm)	D_p _(nm)	S_BET_ (m^2^/g)	V_t_ (cm^3^/g)	Total acidity ^f^ (mmol NH_3_/g)
SAZ-50	50	56	11.2	12.9	9.1	411	1.19	0.19
SAZ-30	30	35	11.2	12.9	9.1	367	1.03	0.24
SAZ-20	20	23	11.1	12.8	9.2	342	0.99	0.32
SAZ-10	10	16	11.6	13.3	-	262	1.02	0.21
Al-SBA-15 ^b^	30	45	10.2	11.7	7.2	466	0.93	0.18
H-ZSM-5 ^c^	30	26	-	-	-	361	0.17	1.15

d_100_: basal spacing; a_0_: unit cell parameter (a_0_ = 2 × d_100_/3^1/2^); D_p_: pore diameter; V_t_: total pore volume; the missing parameters are due to the absence of an ordered mesostructure; ^a^ The number in the SAZ-sample description denotes the initial Si/Al ratio; ^b^ Synthesized by the same procedure for SAZ materials, but using conventional silica sources instead of ZSM-5 nanoseeds; ^c^ Synthesized following the patent of Kulkarni *et al.* [12]; ^d^ In the initial gel mixture; ^e^ In the final product; ^f^ TPD-NH_3_.

**Figure 1:**
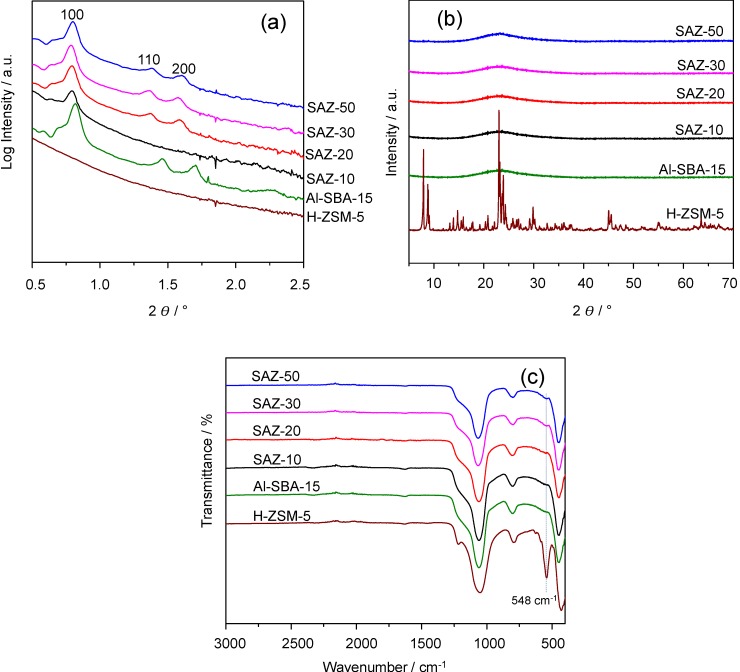
(**a**) Small angle X-ray Scattering (SAXS); (**b**) X-ray diffraction (XRD) patterns; and (**c**) Fourier transform infrared (FTIR) spectra of SAZ materials, Al-SBA-15 and H-ZSM-5.

Figure 1a illustrates the small angle X-ray Scattering (SAXS) patterns of SAZ materials assembled from ZSM-5 nanoseeds with different initial Si/Al ratios and those of Al-SBA-15 and H-ZSM-5. It can be seen that samples SAZ-50, SAZ-30 and SAZ-20, prepared from ZSM-5 precursors having the initial Si/Al of 50, 30, and 20, respectively, exhibit three well-resolved peaks indexed as (100), (110) and (200) reflections of an ordered 2D hexagonal structure with a p6mm symmetry, which is typical of SBA-15 type materials. However, when reducing the initial Si/Al ratio to 10 (SAZ-10), only one reflection (100) can be observed, indicating the formation of a disordered mesostructure. For Al-SBA-15, the same three reflections with higher intensity are visible, suggesting a better ordering of its hexagonal mesostructure than that of SAZ materials. The unit cell parameter a_0_ (calculated from the d_100_ spacing) of SAZ samples is almost unchanged with the lowering of the initial Si/Al ratio from 50 to 20 (a_0_ = 12.8–12.9 nm), but is considerably higher at the initial Si/Al ratio = 10 (a_0_ = 13.3 nm) (Table 1). Al-SBA-15 exhibits the smallest unit cell of 10.2 nm among others, possibly due to the different nature of zeolite nanoseeds and conventional silica based precursors. According to Han *et al.* [6], ZSM-5 nanoseeds containing zeolite building units have stronger rigidity and larger volume than non-structured silica based species. Thus, the self-assembly of ZSM-5 nanoseed precursors with the template appears more difficult and requires more space to connect each other compared to the self-assembly of conventional silica species, which leads to the lesser ordering and larger unit cell of SBA-15 analogs.

The nature of the framework of SAZ materials and reference samples Al-SBA-15 and H-ZSM-5 was investigated by X-ray diffraction (XRD) and Fourier transform infrared (FTIR) spectroscopy. As can be seen from Figure 1b, there are no diffraction reflections in the wide angle XRD pattern of Al-SBA-15, confirming its X-ray amorphous nature. In contrast, H-ZSM-5 displays well-resolved diffraction reflections, characteristic of a highly crystalline ZSM-5. For SAZ samples, the absence of any resolved characteristic diffraction peaks in the wide angle XRD patterns suggests that either their mesostructures are still amorphous at the atomic level or the preserved zeolite building units in their mesopore walls have not connected together to form the long range atomic order of a zeolite framework that can be detected by XRD. To give more insights, FTIR was employed since it is a well-known technique for the identification of zeolite building units in aluminosilicates (Figure 1c) [6,13]. Generally, all SAZ samples exhibit a small band at *ca.* 548 cm^−1^, indicative of five-membered ring subunits of ZSM-5 present [13]. Notably, this band looks more obvious for SAZ-30 and SAZ-50, suggesting a better atomic order for the SAZ samples prepared from lower aluminum containing ZSM-5 nanoclusters. In fact, SAZ-10 and Al-SBA-15 show a quite similar FTIR spectrum, signifying that the ZSM-5 subunits are hardly present in the mesopore walls of SAZ-10. On the other hand, H-ZSM-5 shows a sharp absorption peak at *ca.* 542 cm^−1^, indicative of a much higher degree of zeolite crystallinity, in line with the result of XRD. These observations can be rationalized by considering the fact that the formation of zeolite precursors and their evolution to zeolite crystals decline with increasing aluminum content in the initial gel mixture [14]. Hence, at a high aluminum concentration, it is likely that only few ZSM-5 nanoseed precursors containing zeolite building units are formed and thereby the nearly complete absence of ZSM-5 subunits in sample SAZ-10 is explained.

**Figure 2:**
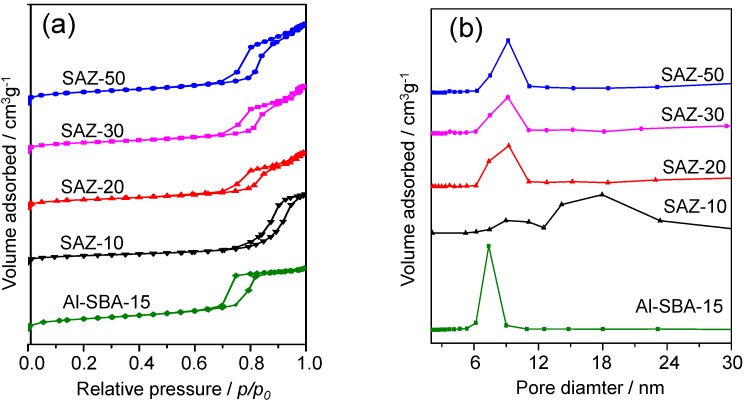
(**a**) N_2_ sorption isotherms; and (**b**) corresponding pore size distributions of SAZ and Al-SBA-15 materials.

The textural properties of SAZ solids, Al-SBA-15 and H-ZSM-5 were studied by N_2_ adsorption/desorption. The N_2_ sorption isotherms and corresponding pore size distribution curves of SAZ and Al-SBA-15 samples are presented in Figure 2a,b, respectively. Detailed textural parameters of all samples are summarized in Table 1.

In line with SAXS results, all SAZ samples exhibit a type IV isotherm with H1-type hysteresis loop, confirming their mesoporous nature. The samples with higher Si/Al ratios (SAZ-50, SAZ-30 and SAZ-20) show a steep capillary condensation step occurring at the relative pressure (*p/p_0_*) range of 0.7–0.9, characteristic of an ordered mesoporous solid with large and uniform pore size. Lowering the initial Si/Al ratio to 10, the hysteresis loop of the isotherm of SAZ-10 becomes irregular and shifts to higher relative pressures, suggesting that its pore uniformity is much degraded. The uniformity and dimension of mesopores can be directly evaluated by their pore size distributions (Figure 2b). Sample SAZ-10 shows a broader pore size distribution ranging from 8 to 24 nm while SAZ-50, SAZ-30 and SAZ-20 samples yield a narrow pore size distribution centered at *ca.* 9.1 nm. The disordered mesostructure of SAZ-10 can be also evidenced by the lowest BET surface area (262 m^2^/g) in relation to that of the other SAZ samples (342–411 m^2^/g) (Table 1). Compared to SAZ samples, the higher ordering of the mesostructure of Al-SBA-15 is further proved by its better defined hysteresis loop and narrower pore size distribution (Figure 2a,b, respectively). On the other hand, the microporous nature of H-ZSM-5 is proven by the type I isotherm with sharply increased nitrogen update at *p*/*p*_0_ < 0.01 (not shown). More details of the textural properties of the reference samples are given in Table 1.

Thus, it is evidenced that the mesostructural ordering of SAZ materials decreases with reducing the initial Si/Al ratio of ZSM-5 nanoseeds. In other words, a high aluminum concentration in the initial gel is not favorable for the formation of an ordered mesostructure. This behavior can be explained based on the synthesis conditions applied in this study. It is generally accepted that the ordered mesostructure of SAZ materials (SBA-15 analogs) is formed by the self-assembly of ZSM-5 precursors with the P123 polymer as a structure directing agent in strongly acidic media at low temperature (1.6 M HCl, 313 K). Under such strongly acidic conditions, it is likely that only part of aluminum, which is presumed to be fixed in the zeolite building units, can be introduced into the mesoporous framework of SBA-15 analogs [6]. The remaining fraction of aluminum still exists in the cationic form that is unlikely to be incorporated in the mesopore walls. Indeed, MAS-9 prepared from ZSM-5 precursors in strongly acidic media gave a relatively low aluminum content, e.g., the Si/Al ratio in the initial gel of 60 resulted in a product with Si/Al = 256 [7]. Therefore, in order to introduce more aluminum in SBA-15 analogs, in this work, the pH of the synthesis mixture was adjusted to 3.5 before hydrothermal treatment at high temperature (473 K) for further silica condensation. At this pH value (mildly acidic media), most of aluminum atoms change into their corresponding oxo species and undergo further condensation with surface silanol groups of the preformed mesostructure, forming Si–O–Al linkages [15,16]. The incorporated aluminum on the surface of mesopores tends to form a layer that can play a protecting role by repelling the attack of water, thus hindering the hydrolysis of Si–O–Si surface bonds, finally preventing the mesostructure from degradation in boiling water. However, Li *et al.* [17] found out that when a large amount of aluminum was introduced into the mesoporous framework, a large fraction of octahedrally-coordinated aluminum species was formed. Under severe hydrothermal treatment, these aluminum species are easily aggregated; thus, the protecting layer is damaged. As a result, the mesostructure of SAZ-10 has partially collapsed during hydrothermal treatment at 473 K.

**Figure 3:**
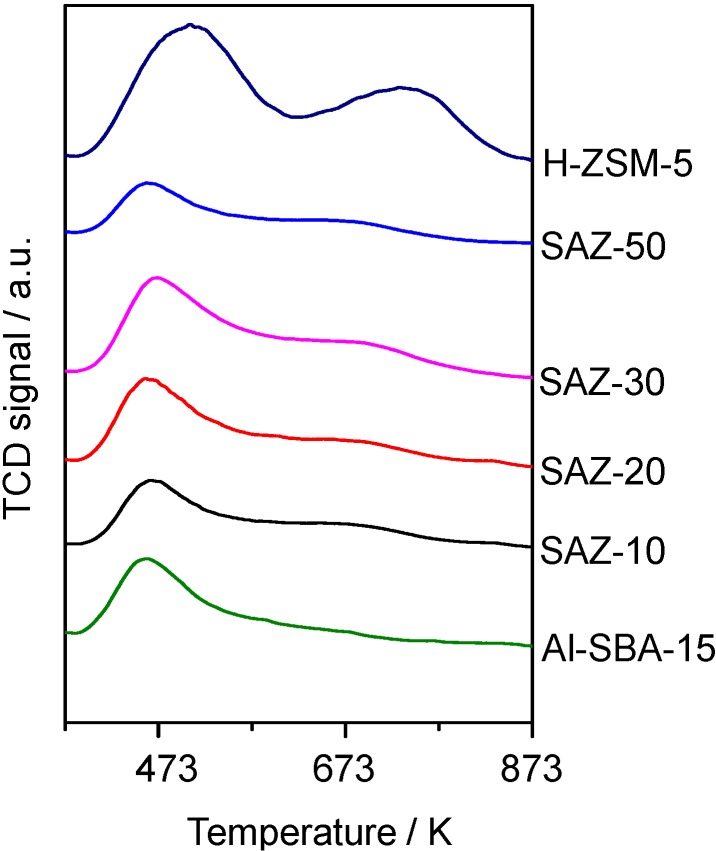
Temperature-programmed desorption of ammonia (NH_3_-TPD) profiles of SAZ solids compared to reference samples Al-SBA-15 and H-ZSM-5.

The number and strength of acid sites of SAZ solids and reference samples Al-SBA-15 and H-ZSM-5 were studied by temperature-programmed desorption of ammonia (NH_3_-TPD). The results are depicted in Figure 3 and Table 1.

It can be seen from Figure 3 that all SAZ materials yield a similar TPD profile with two desorption peaks. The dominant peak at *ca.* 473 K corresponds to weak acid sites and the second broader peak like a tail in the range of 573–773 K indicates the presence of medium and strong acid sites. For the reference samples, the second peak of H-ZSM-5 looks well-developed while that of Al-SBA-15 is hardly visible. This implies that the acidity of SAZ solids is weaker than H-ZSM-5, but stronger than conventional Al-SBA-15.

The number of acid sites estimated from the peak area in the TPD profiles is listed in Table 1. It is apparent that the total acidity of SAZ samples increases with increasing the incorporated aluminum content (SAZ-20 > SAZ-30 > SAZ-50). However, the acid site density of SAZ-10 (0.21 mmol NH_3_/g) is noticeably lower than that of SAZ-20 (0.32 mmol NH_3_/g) although the former solid contains a higher incorporated aluminum content. The reason might be the fact that the high incorporated aluminum content causes the formation and aggregation of octahedrally coordinated aluminum species, which decrease the density of acid sites [17]. On the other hand, it should be noted that at the same initial Si/Al ratio of 30, the acid site density of SAZ-30 is higher than that of Al-SBA-15. This result further supports the work of Han *et al.* [6] and others [7,8,9,18] who found that the introduction of zeolite building units in the mesopore walls induced an improved acidity of SBA-15 analogs. 

### 2.2. Hydrothermal Stability Test

**Figure 4:**
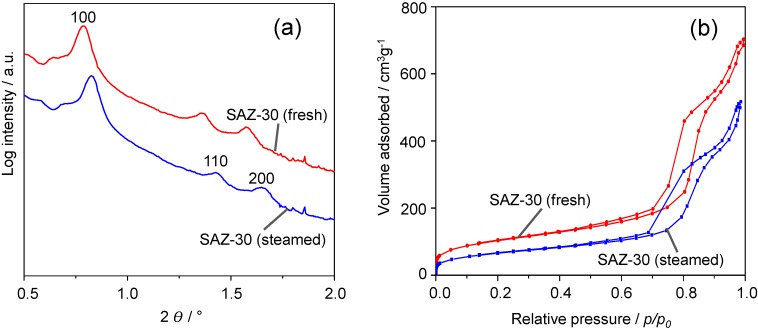
Effect of steaming on the structural and textural properties of SAZ-30: (**a**) SAXS patterns, (**b**) N_2_ sorption isotherms.

**Figure 5:**
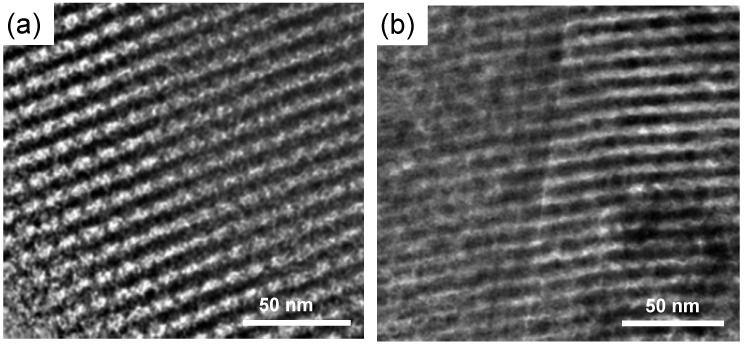
TEM images of (**a**) fresh and (**b**) steamed SAZ-30 viewed along [110] direction.

Hydrothermal stability is of crucial importance as potential catalysts are targeted for the fluid catalytic cracking (FCC) process, wherein the catalyst has to withstand severe conditions, *i.e.*, 973–1073 K in the presence of steam during regeneration step [2]. Therefore, the hydrothermal stability was evaluated by treatment of the representative sample SAZ-30 (1073 K, 30% of steam in 30 mL/min He flow) and subsequent SAXS, N_2_ sorption, and TEM analyses. The results for the fresh and steamed samples are shown in Figure 4, Figure 5 and Table 2.

**Table 2:** Effect of steaming on structural and textural properties of SAZ-30.

SAZ-30	*D*_p_ (nm)	*V*_t_ (cm^3^/g)	*V*_t_ decrease, %	*S*_BET_ (m^2^/g)	*S*_BET_ decrease (%)
Fresh	9.1	1.03	-	367	-
Steamed	9.1	0.85	19.4	263	28.3

*D*_p_: pore diameter; *V*_t_: total pore volume.

Figure 4a shows the SAXS patterns of fresh and steamed SAZ-30. Upon steaming at 1073 K for 24 h, SAZ-30 still displays three main reflections indexed as (100), (110) and (200) planes of the ordered hexagonal symmetry. The remarkable stability of SAZ-30 against steaming is further supported by N_2_ adsorption/desorption studies (Figure 4b). They reveal a similar shape of the adsorption and desorption isotherms of fresh and steamed SAZ-30. Thus, SAXS and BET results suggest the preservation of the SAZ-30 ordered mesostructure. The pore volume and BET surface area of SAZ-30 decrease by about 19% and 28%, respectively, whereas during steaming under the same conditions, the ordered mesostructure of SBA-15 partially collapsed already after 4 h, and the pore volume and BET surface area dropped by 60% and 65% respectively [9]. The mesostructure preservation upon steam treatment is of practical importance because the future FCC process is likely to operate with very short contact time (less than 2 s) [19] where large pores ease molecular transport, and consequently improve catalytic performance.

The high steaming stability is finally confirmed by transmission electron microscopy (TEM) analysis. The TEM image of fresh SAZ-30 (Figure 5a) taken along a direction perpendicular to the pore axis reveals well-ordered hexagonal arrays with large uniform pores. Upon the steam treatment, the mesostructure of SAZ-30 apparently looks degraded, but the regularity of its mesopores is clearly evidenced (Figure 5b). It was reported that there are many factors such as “zeolite-like” connectivity, high temperature synthesis or the salt effect which are favorable for the hydrothermal stability of mesoporous materials [5,8,9,20,21]. Taking these factors into account, the remarkable steaming stability of SAZ-30 can be attributed to the retention of zeolite building units and highly condensed mesopore walls caused by high temperature synthesis.

### 2.3. Catalytic Cracking of Cumene

**Figure 6:**
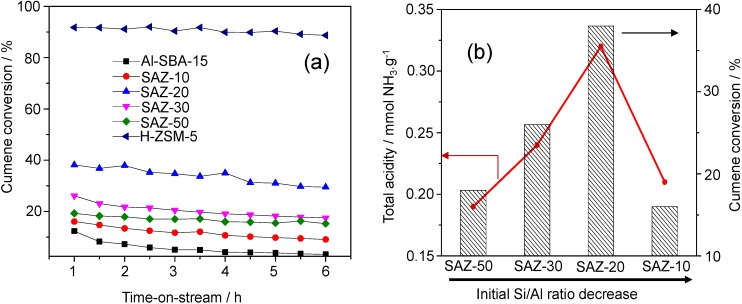
(**a**) Cumene conversion over SAZ materials compared to Al-SBA-15 and H-ZSM-5 as a function of time-on-stream; (**b**) cumene conversion over SAZ materials after 1 h on-stream and their total acidity plotted against the initial Si/Al ratio.

The gas phase cracking of cumene was carried out as probe reaction to evaluate Brønsted acidity of SAZ solids compared to that of conventional Al-SBA-15 and H-ZSM-5. To reach this goal, cumene cracking over SAZ solids, Al-SBA-15 and H-ZSM-5 was run at low temperature (573 K) and a weight hourly space velocity (WHSV) of 1.5 h^−1^ under ambient pressure. The effect of thermal cracking was checked with inert material (glass beads) and found to be negligible. Under the chosen conditions in the presence of a catalyst in this study, cumene was mainly dealkylated to form benzene as a main product, indicating that Brønsted acidity is at play in this reaction [22]. The cumene conversion over various SAZ catalysts, Al-SBA-15 and H-ZSM-5 with time-on-stream is depicted in Figure 6a and the cumene conversion upon 1 h on-stream in relation with their acid site density and initial Si/Al ratios is shown in Figure 6b.

From Figure 6a, it is obvious that H-ZSM-5 and Al-SBA-15 show the highest and lowest cumene conversions, respectively, and SAZ catalysts display cumene cracking activities in between. The highest conversion of cumene over H-ZSM-5 can be attributed to the highest density and strength of acid sites, in particular Brønsted acid sites [1,2]. In contrast, most of incorporated aluminum in amorphous Al-SBA-15 appears to contribute to Lewis acidity due to a lack of a long-range atomic order [16]. Since Lewis acidity is apparently little active under the mild cracking conditions in this study, the lowest cumene conversion over Al-SBA-15 among others has been obtained. The SAZ catalysts, for instance, SAZ-50, have a similar density of acid sites compared to that Al-SBA-15 but exhibit a higher cumene conversion, which further supports the conclusion that the incorporation of zeolite building units in the mesopore walls results in an improved number of acid sites, particularly of active Brønsted acid sites [6,7,8,9].

The effect of the initial Si/Al ratio of ZSM-5 nanoseeds on the catalytic activity of SAZ catalysts is illustrated in Figure 6b. It can be seen that the cumene conversion increases from 18.2% to 38.1% with reducing the initial Si/Al ratio from 50 to 20. Within this initial Si/Al ratio range, a good correlation between the number of acid sites and cumene conversion has been observed. However, adding more aluminum to the initial gel (Si/Al = 10), the acidity and cumene cracking activity of SAZ-10 drop sharply, due possibly to the partial collapse of its mesostructure and the aggregation of octahedral aluminum as above discussed. Thus, in order to obtain an ordered mesostructure of SBA-15 analogs with the enhanced density of acid sites, particularly Brønsted acid sites and high hydrothermal stability, the initial Si/Al ratio of ZSM-5 precursors should not be lower than 20.

## 3. Experimental Section

### 3.1. Chemicals

The chemicals used in this study were tetraethyl orthosilicate (TEOS, 99%, Sigma-Aldrich Chemie GmbH, Taufkirchen, Germany), tetrapropylammonium hydroxide (TPAOH, 20% in water, Sigma-Aldrich Chemie GmbH), aluminum isopropoxide (AIP, 98%, Sigma-Aldrich Chemie GmbH), triblock copolymer pluronic P123 (EO20PO70EO20, MW = 5800, Aldrich, Taufkirchen, Germany), hydrochloric acid (HCl, 37%, Mallinckrodt Baker B.V., Deventer, The Netherlands), ammonium hydroxide (NH_4_OH, 25%, Acros-Organics, Geel, Belgium) and cumene (98%, Sigma-Aldrich Chemie GmbH). All reagents were used as received without further purification.

### 3.2. Synthesis

The preparation of SBA-15 analogs from ZSM-5 nanoseeds involves a two-step process, being similar to that of the previous work [9]. Typically, 6.0 g of TEOS, 10.0 g of TPAOH, 2.0 g of distilled H_2_O were mixed at room temperature and stirred overnight for complete hydrolysis. Then the amounts of AIP required to obtain the Si/Al molar ratio in the range of 10–50 were added and stirred for 24 h, followed by aging at 363 K for 6 h in a reflux system to yield the desired ZSM-5 precursors. 

The P123 solution was prepared by dissolving 2.0 g of P123 in 75 mL of 1.6 M HCl at room temperature for 4 h to get a clear solution. Then, the ZSM-5 precursors solution prepared as described above was added dropwise to the P123 solution, followed by aging at 313 K for 24 h to form an ordered mesostructure in strongly acidic media. Before transferring the mixture into a Teflon-lined autoclave for hydrothermal treatment at 473 K for 24 h, the pH value was adjusted 3.5 with aqueous NH_3_ solution. The final product was filtered off, washed with distilled water, and dried at 373 K for 12 h. The as-synthesized material was calcined in air at 823 K for 5 h with a heating rate of 2 K/min to remove the organic template. The calcined sample is denoted as SAZ-x, where SAZ stands for SBA-15 analogs assembled from ZSM-5 nanoseeds, x is the initial Si/Al molar ratio used in the gel mixture.

For comparison purposes, Al-SBA-15 was synthesized by the same procedure used for the preparation of SAZ, but using conventional silica based sources instead of ZSM-5 nanoseeds. H-ZSM-5 was prepared according to the patent of Kulkarni *et al.* [12]. 

### 3.3. Characterization

SAXS measurements were carried out using a Kratky-type instrument (SAXSess, Anton Paar GmbH, Graz, Austria) operated at 40 kV and 50 mA in slit collimation using a two-dimensional CCD detector. The 2D scattering pattern was converted into a one-dimensional scattering curve as a function of the magnitude of the scattering vector *q* = (4π/λ)sin(θ/2) with SAXSQuant Software (Anton Paar GmbH). A Göbel mirror was used to convert the divergent polychromatic X-ray beam into a collimated line-shaped beam of Cu K radiation (λ = 0.154 nm). Slit collimation of the primary beam was applied in order to increase the flux and to improve the signal quality. The sample cell consisted of a metal body with two windows for the X-ray beam. The powdered samples were sealed between two layers of Scotch^®^ Magic tape (3M France, Cergy Pontoise, France). Scattering profiles of the mesoporous materials were obtained by subtraction of the detector current background and the scattering pattern of the Scotch^®^ tape from the experimental scattering patterns. Correction of instrumental broadening effects (smearing) was carried out with SAXSQuant software (Anton Paar GmbH) using the slit length profile determined in a separate experiment.

X-ray diffraction (XRD) measurements were carried out on a theta/theta diffractometer (X’Pert Pro from PANalytical B.V., Almelo, The Netherlands) with CuKα radiation (λ = 0.15418 nm; 40 kV, 40 mA) and an X’Celerator RTMS Detector. The alignment was checked by use of a silicon standard.

The FTIR spectra were recorded on an ALPHA-FTIR spectrometer (Bruker Optik GmbH, Ettlingen, Germany) using the attenuated total reflection (ATR) sampling technique. 

Nitrogen physisorption measurements were carried out at 77 K on an ASAP 2010 (Micromeritics GmbH, Aachen, Germany). Before measurements, the samples were degassed at 453 K in vacuum for 10 h. The BET specific surface area was calculated using adsorption data at a relative pressure (*p/p*_0_) of 0.05–0.25, and the total pore volume was estimated from the amount adsorbed at a relative pressure of about 0.976. The pore size distributions were obtained from the desorption branch of the isotherm using the corrected form of the Kelvin equation by means of the Barrett-Joyner-Halenda method with a cylindrical pore model.

The TEM measurements were performed at 200 kV on a JEM-ARM200F (JEOL (Germany) GmbH, Eching, Germany) which is aberration-corrected by a CESCOR (CEOS) for the scanning transmission microscopic (STEM) applications. The sample was deposed on a holey carbon supported Cu-grid (mesh 300) as received and transferred to the microscope.

NH_3_-TPD experiments were carried out in a quartz tube reactor in the range of 373–823 K. The liberated ammonia was continuously detected by a thermal conductivity detector (TCD, GOW-MAC Instrument Co., Bethlehem, PA, USA). The sample was first activated at 823 K for 0.5 h under helium flow. After the reactor cooled to 373 K, the sample was swept with helium gas containing 5 vol% of ammonia for 0.5 h for adsorption. Then the feed gas was switched to helium to remove physisorbed ammonia until the TCD baseline was flat. After that, the temperature was increased to 823 K with a heating rate of 10 K/min, and desorbed ammonia was quantitatively analyzed by the external standard method based on comparison of the desorption peak area with the standard curve.

The Al and Si contents were determined by ICP-AES (715-ES, Varian, Inc., Palo Alto, PA, USA) and AAS (AAnalyst 300, PerkinElmer, Inc., Waltham, MA, USA), respectively. For this purpose, the samples were digested with a mixture of HCl-HNO_3_-HF in a microwave-assisted sample preparation system (Multi wave PRO, Anton Paar GmbH) at 473 K and 60 bar. 

### 3.4. Hydrothermal Stability Test

The hydrothermal stability of the representative sample SAZ-30 was evaluated by steaming at 1073 K with 30% water vapor in helium flow (30 mL/min) for 24 h. The sample was sieved and 0.2 g with a particle size between 300 and 700 µm was loaded into a quartz tube reactor. The sample was heated to 1073 K under helium flow with a rate of 20 K/min. As the temperature reached to 1073 K, the helium flow containing 30% steam was introduced into the reactor. The steam treatment time was 24 h. After the steam treatment, the sample was cooled down and characterized by TEM, SAXS and nitrogen physisorption to re-evaluate the structural and textural properties.

### 3.5. Gas Phase Cracking of Cumene

The gas phase cracking of cumene was carried out in a fixed-bed down-flow stainless steel reactor (10 mm internal diameter) equipped with mass flow controllers for reactant dosing. In a typical run, 0.4 g of the sieved catalyst, particle size 300–700 µm, diluted with 2.0 g of quartz beads of the same size was placed in the reactor. Prior to the reaction, the sample was activated in N_2_ flow at 573 K for 2 h to remove physically adsorbed water. Liquid cumene was fed at a rate of 0.6 g/h by a liquid mass flow controller (Liqui-Flow, Bronkhorst High-Tech B.V., Ruurlo, The Netherlands) coupled to a Controlled Evaporator Mixer (CEM, Bronkhorst), using N_2_ flow (90 mL/min) as a carrier gas. By this way, the reactant was mixed with N_2_ and then evaporated before entering the reactor. The catalytic cracking was conducted at 573 K under atmospheric pressure. Feed and product streams were analyzed with an online gas chromatograph (HP 5890, equipped with a sampling valve, a fused silica capillary column (HP5, 50 m × 0.32 mm × 0.52 µm) and a flame ionization detector). The temperature of the column was held at 323 K for 2 min, then increased to 553 K at a rate of 15 K/min and held for 4 min.

## 4. Conclusions

We have shown that an enhanced density of acid sites, in particular Brønsted acid sites of ordered mesoporous SBA-15 analogs (SAZ materials) can be obtained by reducing the initial Si/Al molar ratio of ZSM-5 nanoseeds down to 20. When using a very low initial Si/Al ratio of 10, a disordered mesoporous SBA-15 analog with the degraded textural properties is formed. For such a high aluminum containing SBA-15 analog, the formation and aggregation of octahedral aluminum species during hydrothermal treatment step at high temperature (473 K) may occur, decreasing the density of acid sites. These findings may provide new opportunities for tuning the density of acid sites of ordered mesoporous SBA-15 analogs by varying the initial Si/Al ratio of ZSM-5 nanoseeds. In addition, these acidic mesoporous materials may open the way for the conversion of bulky molecules.
